# Cerebrovascular insult as a consequence of poor health behaviour in patients with schizophrenia and bipolar disorder

**DOI:** 10.1192/j.eurpsy.2024.632

**Published:** 2024-08-27

**Authors:** M. Arnautovic Tahirovic, M. Zuko, I. Lokmic Pekic, N. Sirucic, A. Tahirovic

**Affiliations:** ^1^ Psychiatric hospital of Canton Sarajevo; ^2^Psychiatric clinic, Clinical centre university of Sarajevo, Sarajevo, Bosnia and Herzegovina

## Abstract

**Introduction:**

Cerebrovascular insult (CVI) in patients with psychiatric diseases is to a large extent more prevalent and is accompanied by a worse prognosis after a incident. Despite the higher mortality, these patients are less frequently subjected to CT angiography and interventional intervention on the blood vessels of the brain.

**Objectives:**

To show the frequency of cerebrovascular insults in patients with schizophrenia (SCH), bipolar affective disorder (BP), and depression, depending on age, gender, socioeconomic characteristics, professional qualifications, and dietary habits.

**Methods:**

A total of 1200 patients with SCH, BD and depression were treated over a period of five years.

**Results:**

11.1% SCH patients had CVI and 3,7% a cases of bipolar afective diseases. CVI was most often experienced by patients who were married, employed, or retired, and who lived in urban areas. Smoking, elevated blood pressure values, elevated BMI do not have a significant impact on the occurrence of CVI in all groups. Patients with elevated values of glucose, total cholesterol and LDL cholesterol and CRP had a higher incidence of cerebrovascular insult.

**Conclusions:**

It is necessary to work on raising the awareness of people suffering from psychiatric diseases regarding lifestyle and eating habits, and to conduct periodic health examinations. It is important to recognize high-risk patients and educate them about preventive measures.

**Disclosure of Interest:**

None Declared

